# Efficacy and safety in the short-to-intermediate term of advanced combination therapy with upadacitinib for refractory Crohn’s disease: real-world evidence from eastern China

**DOI:** 10.3389/fmed.2026.1725922

**Published:** 2026-02-17

**Authors:** Zhen-yu Ye, Yang Huang, Ya-nan Wang, Xiang-su Li, Xin Wang, Xu-dong Wu

**Affiliations:** 1 School of Clinical Medicine, Chengdu University of Traditional Chinese Medicine, Chengdu, Sichuan, China; 2 Department of Gastroenterology, Yancheng No.1 People’s Hospital, Yancheng, Jiangsu, China; 3 Department of Gastroenterology, Zhenjiang First People’s Hospital, Zhenjiang, Jiangsu, China; 4 Department of Gastroenterology, Yancheng Clinical College of Xuzhou Medical University, Yancheng, Jiangsu, China

**Keywords:** adverse drug reactions, biological therapy, Crohn’s disease, IBD CLINICAL, upadacitinib

## Abstract

**Aims:**

To evaluate the real-world efficacy and safety of advanced combination therapy with upadacitinib (ACT-U) in East Asian patients with refractory Crohn’s disease (CD).

**Methods:**

This single-center, retrospective, observational study conducted at Yancheng No.1 People’s Hospital (Jiangsu, China) evaluated the efficacy and safety of ACT-U in 26 patients with refractory CD. Participants received ACT-U (upadacitinib + biologics) following inadequate response to ≥1 biologic. Clinical, endoscopic, and biochemical outcomes were assessed at baseline and posttreatment. Safety profiles were monitored throughout the follow-up period to evaluate the risk of adverse events.

**Results:**

Patients (median age 27.5 years [IQR, 23.3–32.0], median disease duration 4.0 years [3.0–6.0]) received ACT-U for a median of 14.5 weeks [11.0–18.8]. Median HBI decreased from 7 [6–8] to 2 [2–4] (*p* < 0.01). All patients achieved clinical response (95% CI: 85.8, 100%), and 91.7% attained remission (95% CI: 73.0, 99.0%). Median SESCD decreased from 15 [13–17] to 1 [0–2], with (*p* < 0.01). All patients achieved endoscopic improvement (95% CI: 85.8, 100%), 83.3% achieved remission (95% CI: 62.6, 95.3%), and 45.8% attained healing (95% CI, 25.6, 67.2%). Mild AEs occurred in 23.1% (95% CI, 9.0, 43.6%; acne, gastrointestinal disturbances, mild infections), including one transient semen discoloration case. No severe AEs or surgeries reported.

**Conclusion:**

ACT-U aligned with the STRIDE-II goals, demonstrating accelerated attainment of short-to-intermediate term endpoints (per STRIDE-II recommendations). Despite limitations, including its retrospective design and small case series, we provide foundational real-world evidence supporting ACT-U as a well-tolerated and effective regimen for refractory CD in EAS patients. Further prospective studies are warranted to validate long-term outcomes, optimize combination strategies, and assess applicability across diverse frameworks.

## Introduction

Crohn’s disease (CD), a primary subtype of inflammatory bowel disease (IBD), has experienced significant shifts in treatment approaches since the 1990s, largely due to the introduction of biologic agents. While these medications have markedly improved clinical outcomes compared with conventional therapies, notable challenges remain: approximately one-third of patients show primary nonresponse to biologics (1), and an additional 20% may experience secondary loss of response within the first year (2). Moreover, there is an annual decline in efficacy of approximately 13% (3). These limitations highlight the ceiling effect of monotherapy regimens (4, 5), which can ultimately result in progressive intestinal complications, such as strictures and penetrating lesions, and may necessitate surgical interventions in cases that are inadequately controlled.

The concept of advanced combination therapy (ACT) is defined by the concurrent use of two biologics with differing mechanisms or the combination of a biologic with a small-molecule agent aimed at bridging existing therapeutic gaps (6). Recent clinical evidence indicates that ACT may improve both clinical and endoscopic remission rates (7). However, the optimal partner agents have yet to be identified as new options continue to emerge in the market.

Upadacitinib (UPA) is a highly selective and reversible oral small molecule Janus kinase (JAK) 1 inhibitor previously used for the treatment of atopic dermatitis, rheumatoid arthritis, and psoriatic arthritis and was recently approved for the treatment of CD and UC. In CD monotherapy, UPA has superior efficacy and tolerability over first-generation JAK inhibitors such as tofacitinib (TFA) (8). Given the higher selectivity of UPA over TFA, UPA may lead to improved outcomes when used in combination regimens, which is an important advantage, compared with the less favorable safety outcomes observed in earlier attempts at TFA-based ACT regimens (9).

Emerging evidence by Yin et al. (10) highlights the promising potential of the UPA-UST combination for the short-term induction of IBD. However, genetic heterogeneity between East Asian (EAS) and European (EUR) populations with CD, for example, the significantly lower allele frequencies of the key risk gene *NOD2* in EAS population, underscore the importance of conducting therapeutic evaluations that are tailored to specific populations (11–13). These differences could have a considerable impact on treatment responses to ACT strategies among different populations.

China’s rapid industrialization and demographic profile position it to bear a disproportionately high share of the global burden of CD in the coming decades (14–16). In light of this, we conducted a retrospective study in Eastern China, a region that exemplifies the epidemiological trends seen in East Asia, to evaluate the novel ACT-U regimen (17). Our study provides preliminary real-world evidence regarding the safety and efficacy in the short-to-intermediate term of ACT-U in treating refractory CD among the EAS population, thereby addressing critical gaps in our understanding of personalized therapy for evolving disease phenotypes.

## Materials and methods

### Study design

This was a single-center, retrospective, observational study conducted in accordance with the Strengthening the Reporting of Observational Studies in Epidemiology (STROBE) guidelines (18). The study protocol was approved by the Medical Ethics Committee of Yancheng No.1 People’s Hospital.

### Patient population and ethics

The retrospective medical record review and the medication regimen involved were approved by the Medical Ethics Committee of Yancheng No.1 People’s Hospital. We included consecutive patients aged ≥18 years with CD who met the following criteria: (1) Received care at the Department of Gastroenterology, Yancheng No.1 People’s Hospital between July 2023 and July 2024; (2) initiated ACT-U, defined as concomitant use of UPA and a biologic agent; and (3) had ≥1 follow-up evaluation posttreatment initiation. In line with the exploratory nature of this real-world study and to maximize case inclusion given the rarity of this therapeutic approach in our setting, no specific exclusion criteria were applied beyond the inclusion criteria listed above. A total of 26 patients were eligible.

Baseline demographic and clinical data, including sex, age, disease duration, Montreal classification parameters (location/behavior), smoking status, prior CD therapies, and surgical history, were extracted from electronic medical records (Table 1). Disease activity was assessed via the Harvey-Bradshaw Index (HBI, Supplementary Table I) (19), serum C-reactive protein (CRP), fecal calreticulin (FC), hemoglobin (Hb), and albumin (Alb) levels at baseline and posttreatment of 12 weeks. Endoscopic activity was quantified via the Simple Endoscopic Score for Crohn’s Disease (SESCD, Supplementary Table II) (20), with assessments performed during standard-of-care endoscopies at corresponding time points, in accordance with the guidelines of the Chinese Medical Association (CMA) (21). Adverse events were monitored throughout treatment and for 8 weeks after discontinuation or the date of the last data capture.

**Table 1:** Demographics and disease characteristics of the patients included.

Characteristics	UPA + biologic (*n* = 26)
Sex	
Male, *n* (%)	18 (69.2)
Female, *n* (%)	8 (30.8)
Median age, years [IQR]	27.5 [23.3–32.0]
Median disease duration, years [IQR]	4.0 [3.0–6.0]
Median treatment duration, weeks [IQR]	14.5 [11.0–18.8]
Disease location, *n* (%)	
L1, Ileal	3 (11.5)
L2, Colonic	1 (3.8)
L3, Ileocolonic	22 (84.6)
L4, Upper GI involved	1 (3.8)
Disease behavior, *n* (%)	
B1, Nonstricturing, nonpenetrating	1 (3.8)
B2, Stricturing	24 (92.3)
B3, Penetrating	1 (3.8)
B2 + B3, Stricturing and penetrating	0 (0)
Perianal fistulizing disease, *n* (%)	1 (3.8)
Age at diagnosis, *n* (%)	
Less than 16 years old	3 (11.5)
17–40 years old	23 (88.5)
Over 40 years old	0 (0)
History of GI surgery, *n* (%)	0 (0)
Prior biologic treatment, *n* (%)	
None	0 (0)
1 biologic	7 (26.9)
2 biologics	17 (65.4)
3 biologics	2 (7.7)
Over 3 biologics	0 (0)
Prior failure of anti-TNF therapy, *n* (%)	26 (100)
Tobacco use, *n* (%)	
Never	22 (84.6)
Former	3 (11.5)
Current	0 (0)
Unknown	1 (3.8)
Concomitant steroids at baseline, *n* (%)	4 (15.4)
Concomitant biologic, *n* (%)	
IFX biosimilar	5 (19.2)
IFX originator	1 (3.8)
ADA	4 (15.4)
UST	7 (26.9)
VDZ	9 (34.6)
Received shingles vaccine, *n* (%)	0 (0)

ADA, adalimumab; GI, gastrointestinal; IFX, infliximab; UPA, upadacitinib; UST, ustekinumab; VDZ, vedolizumab.

### Approach to treatment

ACT-U was implemented as off-label compassionate therapy for patients with refractory CD who had an inadequate response to ≥1 biologic agent approved by the National Medical Products Administration of China (NMPA). The regimen was reserved for individuals with persistent clinical, biochemical, or endoscopic disease activity despite optimized biologic therapy, particularly when disease progression risk warrants dual-pathway targeting. UPA was initiated at an inductive dose of 45 mg once daily while continuing baseline biologic therapy to minimize immunogenicity risks associated with biologic interruption. All patients continued their baseline biologic therapy without interruption, with UPA administered as an add-on treatment. Dose reduction to a maintenance dose of 15 mg once daily was permitted after 8 weeks if clinical remission was achieved per the prescribing provider. A 30 mg maintenance dose was not utilized in this study due to limited local clinical experience with UPA in CD at the time of the study. Considering the risks associated with intensified immunosuppression, no patients received concomitant immunosuppressants (e.g., azathioprine, methotrexate) following the initiation of ACT-U.

### Endpoints

Endpoints were aligned with the Selecting Therapeutic Targets in Inflammatory Bowel Disease 2 (STRIDE-II) recommendations initiated by the International Organization for the Study of IBD (IOIBD) (22). Clinical outcomes: active disease (HBI ≥ 5), response (≥50% reduction in abdominal pain/stool frequency), and remission (HBI < 5 with stool frequency ≤3 and pain score ≤1). Endoscopic outcomes: active disease (SESCD ≥6, isolated ileal disease ≥4), improvement (≥50% SESCD reduction), remission (SESCD ≤3), and healing (SESCD = 0).

### Statistical analysis

Analyses were performed via SPSS 26.0 (IBM Corp.) and R 4.2.3 (R Foundation for Statistical Computing). The normality of continuous data, including the paired differences (post-treatment minus baseline), was assessed using the Shapiro–Wilk test. Continuous variables are presented as medians [IQRs]. For inferential analysis, the median of the paired differences (*Δ*) was calculated as the primary measure of treatment effect. Because the paired differences violated normality assumptions, the non-parametric Wilcoxon matched-pairs signed-rank test was employed for hypothesis testing. To estimate the precision of the median difference, 95% confidence intervals (CIs) were computed using a non-parametric bootstrap approach (bias-corrected and accelerated method, 10,000 replications). Categorical variables are reported as proportions, with intergroup comparisons performed using *χ*^2^ tests. Proportions of binary outcomes are presented with 95% CIs calculated using the Clopper-Pearson exact method.

For patients with multiple post-treatment measurements, the most conservative (i.e., least favorable) value was selected for analysis to minimize the risk of bias. For key efficacy outcomes, 95% confidence intervals (CIs) were calculated where applicable. Scores were excluded if (1) key data were missing, (2) postsurgical anatomy precluded accurate scoring (e.g., HBI in ostomy patients), or (3) baseline activity criteria were unmet. Surgically resected bowel segments were scored as 0 in the SESCD calculations. In accordance with common analytical practices, measurements below the limit of detection (LOD) were substituted with half the LOD value in analyses. Data from participants who discontinued ACT-U treatment were excluded from the per-protocol efficacy analysis.

## Results

### Baseline characteristics and treatment overview

The study included 26 patients with a median disease duration of 4.0 years [3.0–6.0]. Among these patients, 96.2% (25/26) presented with penetrating or stricturing disease (Montreal classification B2/B3), whereas 3.8% (1/26) presented with a history of perianal fistula. No prior gastrointestinal surgeries were reported. A majority (73.1%, 19/26) had failed treatment with ≥2 biologic agents, and all patients (26/26) demonstrated refractoriness to anti-TNF monotherapy. None of the patients received the shingles vaccine prior to treatment initiation due to the current NMPA restrictions on its indications in adults under 50 years of age. ACT-U was initiated on the basis of endoscopic evidence in all patients (26/26), and 1 patient (3.8%) also underwent an intestinal ultrasound (IUS) evaluation. The concomitant biologics included vedolizumab (VDZ, 34.6%, 9/26), ustekinumab (UST, 26.9%, 7/26), infliximab (IFX) biosimilar (19.2%, 5/26), adalimumab (ADA, 15.4%, 4/26), and IFX originator (3.8%, 1/26), with 15.4% (4/26) using concomitant steroids at baseline.

### Clinical and endoscopic outcomes

**Figure 1:**
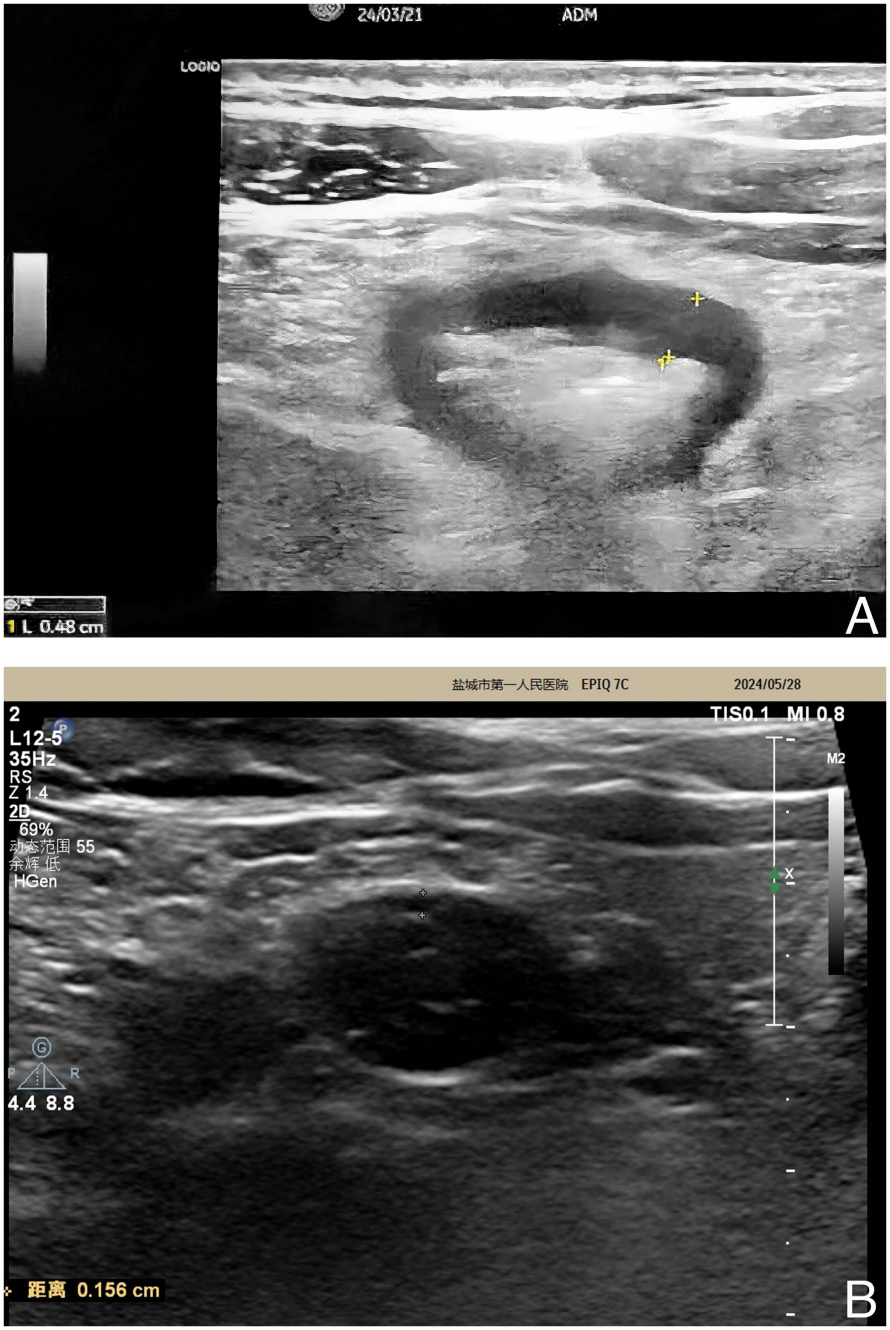
IUS images of the patient. **(A)** The IUS image prior to the initiation of ACT-U demonstrated a maximum BWT of 4.80 mm. **(B)** Following a 9-week regimen of ACT-U, the subsequent IUS image reflects a reduction in maximum BWT to 1.56 mm. This notable decrease suggests a positive therapeutic response to the treatment. ACT-U, advanced combination therapy with upadacitinib; BWT, bowel wall thickness; IUS, intestinal ultrasound.

After a median treatment duration of 14.5 weeks [11.0–18.8], significant improvements in clinical and endoscopic activity were observed. The median HBI decreased from 7 [6–8] at baseline to 2 [2–4] posttreatment, with a median reduction of 4 (95% CI: 3, 4; *p* < 0.01). Clinical response was achieved in all patients (24/24; 95% CI: 85.8, 100%), with 91.7% (22/24; 95% CI: 73.0, 99.0%) attaining clinical remission. Notably, 75.0% (3/4) of patients on concomitant steroids at baseline achieved steroid-free clinical remission. Endoscopic evaluation (*n* = 24) revealed a marked reduction in the median SESCD from 15 [13–17] to 1 [0–2], with a median reduction of 13 (95% CI: 10.5, 13; *p* < 0.01). Endoscopic improvement was observed in all patients (24/24; 95% CI: 85.8, 100%), while 83.3% (20/24; 95% CI: 62.6, 95.3%) achieved endoscopic remission, and 45.8% (11/24; 95% CI: 25.6, 67.2%) attained endoscopic healing. The case received IUS evaluation demonstrated posttreatment normalization, with the maximum bowel wall thickness (BWT) decreasing from 4.80 mm to 1.56 mm and complete resolution of the color Doppler signal (CDS; Figure 1).

### Biomarker changes

Significant biochemical improvements were noted. Inflammatory markers demonstrated marked reductions: CRP levels (*n* = 24) declined from a median of 5.88 mg/L [3.95–8.91] to 1.07 mg/L [0.39–2.15], with a median reduction of 5.00 mg/L (95% CI: 3.79, 6.18; *p* < 0.01), with all (24/24; 95% CI: 85.8, 100.0%) values remaining within normal limits (0–10 mg/L). FC levels (*n* = 24) exhibited a striking decline from a median of 160.46 μg/g [138.67–202.90] to 15.00 μg/g [15.00–36.08], with a median reduction of 134.68 μg/g (95% CI: 109.76, 177.83; *p* < 0.01), achieving normalization (0.00–50.00 μg/g) within 91.7% (22/24; 95% CI: 73.0, 99.0%) of patients. Moreover, hematological and nutritional parameters improved in parallel: Hb levels (*n* = 24) increased from a median of 127 g/L [121–131] to 133 g/L [130–135], with a median increase of 6 g/L (95% CI: 5, 7; *p* < 0.01), and all patients (16/16; 95% CI: 79.4, 100%) who presented baseline anemia approached or achieved normalization (130–175 g/L). Alb levels (*n* = 24) rose from a median of 39.4 g/L [36.5–42.7] to 43.3 g/L [40.8–46.4], with a median increase of 3.25 g/L (95% CI: 2.85, 4.05; *p* < 0.01), restoring normal levels (42.0–56.0 g/L) in 47.1% (8/17; 95% CI: 23.0, 72.2%) of patients with baseline Alb abnormalities.

### Safety and treatment discontinuation

Adverse events (AEs) were reported in 23.1% (6/26; 95% CI: 9.0, 43.6%) of the patients, primarily acne (*n* = 3), gastrointestinal disturbances (*n* = 2), mild infections (*n* = 1), and transient blue discoloration of semen (*n* = 1). No cases of shingles, cardiovascular events, venous thromboembolic events (VTEs), or severe AEs were observed. Two patients (7.7%) discontinued UPA: one due to the evaluation of the effectiveness of transitioning to biologic monotherapy after sustained remission, and one (3.8%, 1/26; 95% CI: 0.1, 9.6%) due to mild infections and transient semen discoloration. No disease-related surgeries or hospitalization events occurred during follow-up.

Key efficacy and safety outcomes are summarized in Tables 2, 3.

**Table 2:** Efficacy and safety outcomes: binary outcomes.

Outcome measure	*n*/*N*	Percentage (%) [95% CI]
*Clinical efficacy*		
Response	24/24	100 [85.8, 100]
Remission (HBI < 5)	22/24	91.7 [73.0, 99.0]
*Endoscopic efficacy*		
Improvement (≥50% SESCD reduction)	24/24	100 [85.8, 100]
Remission (SESCD ≤3)	20/24	83.3 [62.6, 95.3]
Healing (SESCD = 0)	11/24	45.8 [25.6, 67.2]
*Biomarker normalization*		
CRP	24/24	100 [85.8, 100]
FC	22/24	91.7 [73.0, 99.0]
Hb*	16/16	100 [79.4, 100]
Alb*	8/17	47.1 [23.0, 72.2]
*Safety outcomes*		
Any AE	6/26	23.1 [9.0, 43.6]
Treatment discontinuation due to AE	1/26	3.8 [0.1, 9.6]

CI, confidence interval; HBI, Harvey-Bradshaw Index; SESCD, simple endoscopic score for Crohn’s disease; CRP, C-reactive protein; FC, fecal calprotectin; Hb, hemoglobin; Alb, albumin; AE, adverse event. *Calculated only for patients with abnormal baseline values.

**Table 3:** Efficacy outcomes: continuous variables before and after treatment.

Variable	Baseline, Mdn [IQR]	Posttreatment, Mdn [IQR]	Mdn difference [95% CI]
*Clinical activity*			
HBI	7 [6–8]	2 [2–4]	4 [3, 4]
*Endoscopic activity*			
SESCD	15 [13–17]	1 [0–2]	13 [10.5, 13]
*Biomarkers*			
CRP (mg/L)	5.88 [3.95–8.91]	1.07 [0.39–2.15]	5.00 [3.79, 6.18]
FC (μg/g)	160.46 [138.67–202.90]	15.00 [15.00–36.08]	134.68 [109.76, 177.83]
Hb (g/L)	127 [121–131]	133 [130–135]	-6 [−7, −5]
Alb (g/L)	39.4 [36.5–42.7]	43.3 [40.8–46.4]	−3.25 [−4.05, −2.85]

Mdn, median; IQR, interquartile range; CI, confidence interval; HBI, Harvey-Bradshaw Index; SESCD, Simple Endoscopic Score for Crohn’s Disease; CRP, C-reactive protein; FC, fecal calprotectin; Hb, hemoglobin; Alb, albumin.

## Discussion

### Promising outcomes of the ACT-U: allied with the STRIDE-II goals

STRIDE-II (22) establishes both short-term and intermediate-term goals for CD treatment. During the previous era of biologic monotherapy, the mid-term success rate of the STRIDE-II, as well as its short-term success rate, was not satisfactory. According to findings from phase III clinical trials, the short-term success rate (remission rate during the induction phase) for biologic monotherapy ranged from only 14.5 to 36% (23–26). If not effectively controlled, patients with CD may experience additional complications, such as stricturing or penetration of the gastrointestinal tract, which may ultimately require surgical intervention (27, 28). While there have been previous attempts at combination therapies, the promising experimental results from these attempts have not been widely adopted as standard clinical practices (29, 30). The ECCO 2024 updated guidelines (31) have further brought attention to ACT regimens.

This study revealed that ACT-U is a well-tolerated and effective ACT regimen for improving the clinical performance of patients and reducing CRP and FC levels. These outcomes align with the short- and intermediate-term goals outlined in the STRIDE-II. Notably, ACT-U therapy has demonstrated its ability to help some patients achieve or approach the long-term endoscopic improvement goals of STRIDE-II sooner than anticipated within a median treatment of 15.4 weeks. Accumulating evidence has demonstrated that IUS is a highly sensitive tool for monitoring IBD (32, 33), as acknowledged in STRIDE-II, owing to its capacity to assess disease activity throughout the entire bowel, its repeatability, and its ability to evaluate transmural response and healing in IBD patients. In the case that underwent IUS monitoring in our study, ACT-U resulted in a significant reduction in maximum BWT, with no CDS observed posttreatment, indicating that transmural healing was achieved, in line with the definition put forth by a recent expert consensus statement (34, 35).

### Semen discoloration: should we be concerned?

This study also revealed that the short- to intermediate-term safety of ACT-U in patients with CD is generally not different from the risk profile previously reported in trials of UPA monotherapy (36). Notably, one patient reported a transient blue discoloration of semen, which has not been reported previously. Given that the color change observed was a transient phenomenon rather than a persistent phenomenon, we were unable to conduct further tests on blue semen to identify the underlying cause. *Pseudomonas aeruginosa* (PsA) is a commonly found aerobic-partially anaerobic, gram-negative, conditionally pathogenic bacterium. It is one of the prevalent colonizers of the male urinary tract and frequently causes urinary tract infections, particularly in elderly patients and those with indwelling urinary catheters in clinical settings (37). The metabolism of PsA results in the production of a secondary metabolite known as pyocyanin, which typically appears bluish-green. Its oxidized form can be blue or dark blue under alkaline conditions and reddish under acidic conditions. Since the normal pH of human semen ranges from 7.2 to 8.0, which is slightly alkaline, pyocyanin and its oxidized products are expected to exhibit bluish-green to dark-blue coloration under these circumstances.

Given that both biologics and small-molecule agents are immunosuppressive and carry a risk of opportunistic infections, it is reasonable to consider that urogenital infections caused by PsA might explain the change in semen color observed in this case (38). If further evidence indicates that this change is indeed a manifestation of an opportunistic infection, then the safety profile of the ACT-U regimen may be comparable to that of UPA or biologic monotherapy. However, opportunistic infections arising from the use of biologics and small-molecule drugs are typically persistent, which raises questions as to why the alteration in semen color is only a transient occurrence in this case. PsA has been recognized as a risk factor for male infertility, as it can disrupt sperm function by interfering with sperm capacitation and protein phosphorylation (39–41). Therefore, out of an abundance of caution, it is advisable for male patients undergoing ACT treatment to utilize contraception and to discontinue the drug while seeking symptomatic anti-infective treatment in cases of opportunistic infections.

### Differences in outcomes: possible explanations

In the present study, we observed overall clinical response and remission rates of 100 and 91.7%, respectively, surpassing those reported in previous studies (10, 42). Historically, IBD was perceived primarily as a Western condition. However, data on recent trends (43) indicate a significant increase in the incidence of IBD within newly industrialized countries in the Middle East, Asia, and South America, attributed to rapid industrialization and urbanization over the past two decades. In contrast, the incidence of IBD in developed regions, such as Europe and the United States, has shown relative stability.

These epidemiological variations contribute to a divergent demographic profile of CD patients between emerging and developed nations, with a notable trend toward a younger age group in the former, a finding that aligns with the age distribution of patients enrolled in our study. Prior evidence suggests that patients who experience earlier disease onset generally have more favorable prognoses in response to treatment (44). In our case series, a substantial majority presented with early-onset CD, potentially influencing the characteristics of disease behavior.

Furthermore, a meta-analysis encompassing 25 trials indicated that the initiation of biological therapy during the early stages of IBD may yield greater efficacy, particularly for CD (45). Given this age-related disparity, Chinese patients, especially those residing in Eastern China, where medical conditions are relatively conducive, are ideally positioned to access advanced therapeutic options at a younger age and with a shorter duration of onset. This factor may contribute to an increased response rate to combination regimens.

Recent clinical discourse has introduced the strategy “hit hard and early” with “top-down,” which stands in contrast to the traditional sequential ascending step approach (46, 47). Available evidence supports the notion that this strategy can achieve superior treatment outcomes (47). Our inclusion of patients who experienced fewer overall biologic monotherapy failures, coupled with a relaxed definition of treatment failure for the last time, shifting from loss of response to incomplete response, facilitated more timely transitions to ACT regimens. This alignment with the “hit hard and early” philosophy may have further augmented overall patient success rates.

Moreover, choosing a rational combination is also very important for the treatment’s prognosis. Past experience suggests that applying a combination of complementary mechanisms of action and minimal cascade or cross-talk interactions may help increase activity (48). Given that both UST and UPA can target JAK-related pathways, the combination of these two methods may prove less effective than other combinations that involve different mechanisms, as indicated by previous small-scale reports (10, 42). In our study, the proportion of concomitant biologics based on complementary mechanisms was notably greater, which may also contribute to our superior effectiveness results.

### Beyond the initial results: what else to consider?

ACT was conceived with various flexible coadministration models to cater to diverse clinical scenarios (5). Potential strategies for initiating an ACT regimen include simultaneous coinduction, add-on therapy, and bridge/sequential therapy. The simultaneous coinduction approach aligns with the recently advocated “top-down” strategy (47), which aims to enhance therapeutic outcomes from the very beginning of treatment while mitigating the risk of disease progression. This method may be particularly advantageous for patients newly diagnosed with IBD, positioning it as a potential standard practice in the future.

In contrast, add-on therapy and bridge/sequential therapy are more appropriate for patients who have already started biologic therapy. Given that all patients in our study were subjected to add-on therapy during the induction phase, this study does not provide insights into the efficacy of ACT-U when it is implemented under a bridge/sequential therapy framework. Previous studies have demonstrated the potential of bridge/sequential therapy, especially in combinations involving calcineurin inhibitors and VDZ (49–51). However, the applicability of ACT-U in this form warrants further clinical validation.

For patients who have achieved favorable outcomes with ACT therapy, deliberating whether to persist with the combination, withdraw an agent, or adopt a short-term intermittent regimen has emerged as a critical consideration. Considering the inherent risks associated with combination therapies, sustaining efficacy with a single agent may represent a safer strategy, which may involve a gradual tapering approach to withdrawal. Should efficacy decline during such efforts, referral to a short-term intermittent combination may be considered. Those without prior significant adverse effects may further resume a long-term combination regimen to ensure continued efficacy if needed.

Overall, compared with monotherapy, ACT-U has demonstrated superior clinical benefits for patients with refractory CD. However, it is projected that, over the forthcoming decades, developing countries will gradually become the main battleground in the fight against CD. Increasing the accessibility of biologics and small molecules in these regions will be crucial in alleviating the disease burden. This endeavor depends on the future development and promotion of generic medications and the further refinement of health and welfare policies. Personalized therapy has become the cornerstone of future treatment paradigms. Patients deemed suitable for ACT-U should transition to this therapy after comprehensive discussions regarding the associated risks and benefits, taking into account individual genotypes, underlying medical conditions, and prior therapeutic experiences. Close monitoring of efficacy and AEs is essential for ensuring personalized and adaptive treatment adjustments aimed at optimizing therapeutic benefits.

Our study does present certain limitations. First, this was a retrospective, single-center analysis, and the sample size was relatively small. Second, owing to constraints in sample acquisition, we were unable to delineate the specific etiologies of changes in semen color. Third, our evaluation focused exclusively on the add-on therapy framework, thus precluding an assessment of the effectiveness of ACT-U in the context of bridge/sequential treatment. Finally, the limited duration of follow-up may be insufficient to fully assess the long-term efficacy and safety of ACT-U.

## Conclusion

Overall, ACT-U is effective in enhancing clinical, endoscopic, and ultrasound outcomes in EAS patients with refractory CD. The safety profile of this regimen is consistent with previous reports on UPA monotherapy, which primarily revealed mild adverse events, with no severe adverse events reported. The case of transient semen discoloration underscores the need for additional mechanistic investigations, although its clinical significance remains uncertain. Our findings suggest that the ACT-U is a promising approach for overcoming the ceiling effect of monotherapies, particularly in East Asian populations. Nevertheless, larger prospective, multicenter studies are essential to further investigate long-term outcomes, identify optimal combination partners, and assess the applicability of ACT-U across diverse treatment frameworks.

## Data Availability

The raw data supporting the conclusions of this article will be made available by the authors, without undue reservation.
