# Supplementary material for: Efficacy and safety in the short-to-intermediate term of advanced combination therapy with upadacitinib for refractory Crohn’s disease: real-world evidence from eastern China
Source: Front Med (Lausanne). 2026 Feb 17;13:1725922. doi: 10.3389/fmed.2026.1725922 (PMC12954622; doi:10.3389/fmed.2026.1725922)
Supplement: Supplementary file 1 [file Supplementary_file_1.docx]

**Supplementary Materials**

| **Variable** | **Scoring Method^#^** | | | | |
| --- | --- | --- | --- | --- | --- |
|  | **0** | **1** | **2** | **3** | **4** |
| Patient well-being | Very well | Slightly below par | Poor | Very poor | Terrible |
| Abdominal pain | None | Mild | Moderate | Severe | N/A |
| Abdominal mass | None | Dubious | Definite | Definite and tender | N/A |
| Number of liquid or soft stools | Score 1 per movement | | | | |
| Complications^*^ | Score 1 per item | | | | |

**Suppl Table I. Calculating method of Harvey-Bradshaw Index (HBI).** ^*^ Complications: including arthralgia, uveitis, erythema nodosum, aphthous ulcer, pyoderma gangrenosum, anal fissure, appearance of a new fistula, and abscess. ^#^≤ 4: Remission; 5–7: Mild disease; 8–16: Moderate disease; > 16: Severe disease. The current study employed the version (in Chinese) of the CMA guidelines^i^ as recommended.

| **Variable** | **Scoring Method^#^** | | | |
| --- | --- | --- | --- | --- |
|  | **0** | **1** | **2** | **3** |
| Size of ulcers^*^ | None | Aphthous ulcers  Ø 0.1–0.5 cm | Large ulcers  Ø 0.5–2 cm | Very large ulcers  Ø > 2 cm |
| Ulcerated surface^†^ | None | < 10% | 10–30% | > 30% |
| Affected surface^‡^ | Unaffected segment | < 50% | 50–75% | > 75% |
| Presence of narrowings | None | Single,  can be passed | Multiple,  can be passed | Cannot be passed |

**Suppl Table II. Calculating method of Simple Endoscopic Score for Crohn's Disease (SESCD).** ^*^Evaluate the most severe ulcer present in each segment of the intestine. ^†^Determine the percentage of the total ulcer area for each intestinal segment. ^‡^Calculate the percentage of the total lesion area in each intestinal segment, which includes all lesions except for chronic inflammatory polyps that do not exhibit surface erosion or ulcers. ^#^Scores must be recorded individually for each of the five intestinal segments: rectum, descending colon and sigmoid colon, transverse colon (including the splenic flexure), ascending colon (covering the ileocecal region and hepatic flexure), and ileum. An assessment of each intestinal segment is deemed valid only if conducted with a scope that reaches at least 10 cm. If the aforementioned criteria cannot be fulfilled due to surgical resection, intestinal stenosis that hinders endoscopic passage, colonoscopy technique issues, or other factors, the segment will be classified as unscorable. The total score is calculated as the sum of scores from all five intestinal segments. Currently, a total score of 0–2 signifies remission, a score of 3–6 indicates mild activity, 7–15 reflects moderate activity, and a score of ≥16 denotes severe activity.^ii^ The current study employed the version (in Chinese) of the CMA guidelines^ii^ as recommended.

**References**

^i^ Inflammatory Bowel Disease Group of Chinese Society of Gastroenterology of Chinese Medical Association. Chinese Consensus on Diagnosis and Treatment in Inflammatory Bowel Disease (2018, Beijing). *Chin J Dig*. 2018;38(5):292-311. doi: 10.3760/cma.j.issn.0254-1432.2018.05.002. (In Chinese)

^ii^ Inflammatory Bowel Disease Group of Chinese Society of Gastroenterology of Chinese Medical Association, Inflammatory Bowel Disease Quality Control Center of China. Chinese Clinical Practice Guideline on the Management of Crohn’s Disease (2023, Guangzhou). *Chin J Dig*. 2023;44(2):100-132. doi: 10.3760/cma.j.cn311367-20240109-00010. (In Chinese)
